# Profiling safety behavior in clinical laboratory environments

**DOI:** 10.3389/fpubh.2025.1681513

**Published:** 2025-09-26

**Authors:** Vedat Caner

**Affiliations:** Department of Occupational Health and Safety, Vocational School, Istanbul Beykent University, Istanbul, Türkiye

**Keywords:** occupational safety, laboratory behavior, safety behavior, behavioral profiling, clinical diagnostics

## Abstract

**Introduction:**

Laboratory safety behavior is crucial for minimizing risks in high-hazard clinical settings, yet behavioral non-compliance persists as a leading cause of laboratory accidents despite established protocols.

**Methods:**

This study evaluated safety behavior among 92 personnel employed in genetic diagnostic laboratories in Istanbul using a validated 34-item safety behavior scale. Principal component analysis (PCA), multiple linear regression, and k-nearest neighbors (k-NN) classification were employed to analyze the data.

**Results:**

The analysis revealed three underlying behavioral dimensions: personal compliance, proactive behavior, and institutional engagement. Regression analysis indicated that perceived institutional support and the frequency of safety training were significant predictors of overall safety behavior (*R*^2^ = 0.47, *p* < 0.001). Furthermore, the k-NN classifier utilizing PCA-derived components achieved an 88% accuracy rate in distinguishing high and low compliance profiles.

**Discussion:**

These findings underscore the utility of multivariate behavioral analytics in profiling laboratory safety behavior and highlight the potential of data-informed, classification-based strategies to enhance safety interventions. Adopting behaviorally tailored approaches to training and institutional support may markedly improve compliance and mitigate risk in laboratory environments.

## 1 Introduction

Ensuring occupational safety in laboratory environments is a core element of both institutional responsibility and employee wellbeing (1). Genetic diagnostic laboratories represent a particularly sensitive domain, where biological and chemical exposures are not only frequent but also uniquely complex due to the nature of the materials handled (2). In these environments, staff face risks such as direct exposure to pathogenic agents, reagent-related injuries, and cognitive overload from precision-intensive tasks. Despite protocols and safeguards, many accidents still stem from lapses in human behavior (3).

Traditional research in occupational safety has largely emphasized physical measures, technical controls, and regulatory oversight. However, the behavior of workers—including adherence to procedures, reporting unsafe conditions, and consistent use of personal protective equipment (PPE)—has emerged as a central determinant of safety outcomes (4). This is especially critical in laboratory contexts, where small errors can result in contamination, compromised data, or harm to personnel (5).

Although behavioral safety approaches have been applied in industrial and healthcare domains, their integration into genetic diagnostic laboratories remains limited. Many existing studies view laboratory workers as a homogeneous group or rely primarily on external audits. These approaches overlook the underlying behavioral mechanisms that shape safety performance (6–8). Furthermore, research in this field often employs single-variable assessments, which cannot capture the multidimensional nature of safety behavior.

Applied Behavior Analysis (ABA) provides a robust theoretical framework for addressing this gap. ABA emphasizes the systematic study of observable behavior and its interaction with environmental and organizational variables. By focusing on reinforcement, feedback, and context, ABA enables a deeper understanding of how safe practices are learned, maintained, or neglected in high-risk laboratory environments (9). Yet, despite its potential, ABA methodologies remain underutilized in occupational safety research, particularly in genetic diagnostic settings.

The lack of data specific to Turkey further highlights the importance of the present study. Most published research originates from North America and Western Europe, leaving a gap in understanding how cultural, institutional, and resource-related differences influence safety practices in other contexts. By examining genetic diagnostic laboratories in Istanbul, this study addresses this gap while providing insights that are relevant to both local and global audiences.

This research contributes to the international literature by combining applied behavior analysis with advanced statistical and computational methods. Specifically, it applies principal component analysis (PCA) and k-nearest neighbors (k-NN) to classify compliance profiles and investigate the predictive role of institutional support and training. By bridging behavior analysis with machine learning, the study offers a novel, data-driven approach to understanding safety culture.

In summary, this study aims to:

1. Evaluate laboratory workers’ perceptions and behaviors related to occupational safety.
2. Assess the reliability of the safety behavior measurement tool.
3. Identify latent behavioral components using PCA.
4. Examine predictive relationships between institutional support, training, and safety behavior.

This study generates evidence-based insights to inform tailored training protocols and policy interventions. By highlighting both the Turkish context and its international relevance, it contributes to closing a critical gap in the literature on occupational safety.

## 2 Materials and methods

### 2.1 Study design

This study adopted a cross-sectional, descriptive–correlational design grounded in applied behavior analysis principles. The objective was to examine laboratory workers’ safety behaviors using a structured self-report survey combined with multivariate statistical and computational techniques.

### 2.2 Setting and participants

The research was conducted in 11 genetic disease diagnostic laboratories located in Istanbul, Türkiye. These institutions were selected based on active operation under BSL-2 or BSL-3 standards and willingness to participate. A purposive sample of 92 employees was recruited, including laboratory technicians (42%), molecular biologists and clinical geneticists (36%), and administrative or support staff involved in laboratory tasks (22%).

Inclusion criteria were:

- At least 1 year of laboratory experience,
- Direct or indirect exposure to biosafety procedures,
- Voluntary informed consent.

Participants’ ages ranged from 24 to 57 years (*M* = 36.4, SD = 7.2), with 67% identifying as female. Most had bachelor’s or higher degrees in molecular biology, genetics, or biochemistry. Given that the sample was limited to Istanbul, findings should be interpreted with caution when generalizing to broader populations.

### 2.3 Sampling strategy and sample size justification

The purposive sampling strategy was appropriate for the specialized study population. Sample size adequacy was estimated using G*Power 3.1 for multiple linear regression with two predictors, indicating a minimum of 68 participants. The final sample of 92 exceeded this threshold, supporting statistical power.

Kaiser-Meyer-Olkin (KMO = 0.86) and Bartlett’s Test of Sphericity (*p* < 0.001) confirmed sample adequacy for factor analysis. Nonetheless, it is acknowledged that applying PCA to 34 items with a sample of 92 may limit the stability of extracted components compared to larger samples typically recommended in the psychometric literature.

### 2.4 Instruments and measures

A structured questionnaire was developed based on validated occupational safety behavior scales and behavior analytic frameworks. It included:

1. *Demographic/professional background* (e.g., age, gender, education, experience, training).
2. *Perceived institutional support* (eight items, *α* = 0.87).
3. *Safety behavior scale* (34 items, *α* = 0.91) measuring PPE use, reporting unsafe conditions, participation in training, handling of hazardous materials, and adherence to SOPs.

Responses were recorded on a 5-point Likert scale (1 = “Never” to 5 = “Always”). Higher scores indicated stronger compliance with safety behaviors.

Given the self-report format, potential for social desirability bias is recognized as a methodological limitation. To reduce this risk, participation was anonymous and no identifying information was collected.

### 2.5 Variable coding and regression analysis

The dependent variable was the mean score of the 34-item safety behavior scale. Independent variables included perceived institutional support (continuous) and frequency of safety training.

Training frequency was measured on an ordinal scale (1 = never, 2 = once, 3 = twice, 4 = three or more). For regression purposes, it was treated as continuous, assuming approximately equal spacing between categories. This approach is common in behavioral research, though it may reduce precision compared to ordinal regression. The decision was based on preliminary tests confirming linear trends, but the limitation is acknowledged.

### 2.6 Data collection procedure

Data were collected over 4 weeks in Spring 2024 after obtaining Institutional Review Board approval (Approval No: 2024-056). Surveys were distributed digitally or in sealed envelopes by laboratory supervisors. Participation was voluntary, with informed consent obtained.

### 2.7 Ethical considerations

Procedures followed the Declaration of Helsinki. Ethical approval was granted by the affiliated university ethics committee. Data were anonymized and stored securely in encrypted form.

### 2.8 Data analysis strategy

Data analysis was conducted using IBM SPSS v28.0 and cross-validated classification procedures.

- *Descriptive Statistics*: To summarize demographics and behavior frequencies.
- *Reliability Analysis*: Cronbach’s alpha was used to assess scale consistency.
- *Correlation and Regression*: Pearson correlations and multiple linear regression tested the predictive role of institutional support and training on safety behavior. While the regression model explained 47% of the variance (*R*^2^ = 0.47), the unexplained variance highlights the need for additional predictors in future studies.
- *Principal Component Analysis (PCA)*: Used to reduce dimensionality of the 34-item scale. PCA was chosen for its interpretability and suitability for continuous variables. While alternatives such as exploratory (EFA) or confirmatory factor analysis (CFA) may provide stronger theoretical grounding, PCA was preferred as an initial data-reduction method given the exploratory aims of this study. The relatively modest sample size remains a limitation.
- *k-Nearest Neighbors (k-NN)*: Applied to classify safety profiles using PCA-derived dimensions. To mitigate overfitting with the modest sample size, five-fold cross-validation was performed. Performance was evaluated not only with accuracy but also with precision, recall, and F1-scores, offering a more comprehensive view of classifier reliability.

## 3 Results

### 3.1 Descriptive statistics and reliability analysis

The sample included 92 participants with a mean age of 36.4 years (SD = 7.2); 67% were female, and 89% held at least a bachelor’s degree. Average laboratory experience was 9.1 years (SD = 5.3), and weekly working hours averaged 43.2 (SD = 6.1). Most participants (86%) reported prior safety training. Mean perceived institutional support was 3.87 (SD = 0.71), and the overall safety behavior score was 4.14 (SD = 0.66), indicating frequent engagement in safe practices.

The 34-item safety behavior scale demonstrated high internal consistency (*α* = 0.91). PCA-derived subscales also showed strong reliability: Personal Compliance (*α* = 0.89), Proactive Behavior (*α* = 0.84), and Safety Engagement (*α* = 0.86). A summary of descriptive statistics and reliability metrics is presented in Table 1.

**Table 1:** Descriptive statistics and scale reliability.

No.	Measure	Value
1	Age (years)	36.4 ± 7.2
2	Gender (female)	67%
3	Education (bachelor or higher)	89%
4	Laboratory experience (years)	9.1 ± 5.3
5	Weekly working hours	43.2 ± 6.1
6	Received safety training	86%
7	Perceived institutional support (mean ± SD)	3.87 ± 0.71
8	Safety behavior score (mean ± SD)	4.14 ± 0.66
9	Item-total correlation range	0.45–0.78
10	Cronbach’s alpha (overall)	0.91
11	Cronbach’s alpha (personal compliance)	0.89
12	Cronbach’s alpha (proactive behavior)	0.84
13	Cronbach’s alpha (engagement)	0.86

### 3.2 Inferential analysis across demographic subgroups

Independent *t*-tests indicated no significant gender or education differences in safety behavior scores (*p* > 0.05). However, ANOVA revealed significant age effects, with younger workers (24–35 years) reporting lower safety behavior than older groups, *F*(2, 89) = 4.27, *p* = 0.02.

### 3.3 Principal component analysis

- PCA supported a three-component solution explaining 67.4% of variance: Personal Compliance, Proactive Behavior, and Safety Engagement. These components reflected compliance with protocols, proactive risk reporting, and active participation in organizational safety culture.

Item loadings above 0.40 were considered meaningful. The full results are presented in Table 2.

**Table 2:** Principal component loadings for safety behavior items.

Item	Component 1 (personal compliance)	Component 2 (proactive behavior)	Component 3 (safety engagement)
Use of PPE	0.81	0.15	0.12
Hand hygiene	0.77	0.20	0.10
Follow SOPs	0.74	0.25	0.15
Report hazards	0.22	0.73	0.18
Take initiative	0.19	0.70	0.21
Address unsafe acts	0.18	0.68	0.23
Attend safety trainings	0.12	0.24	0.79
Communicate with supervisors	0.09	0.18	0.76
Provide safety feedback	0.11	0.20	0.74

**Figure 1:**
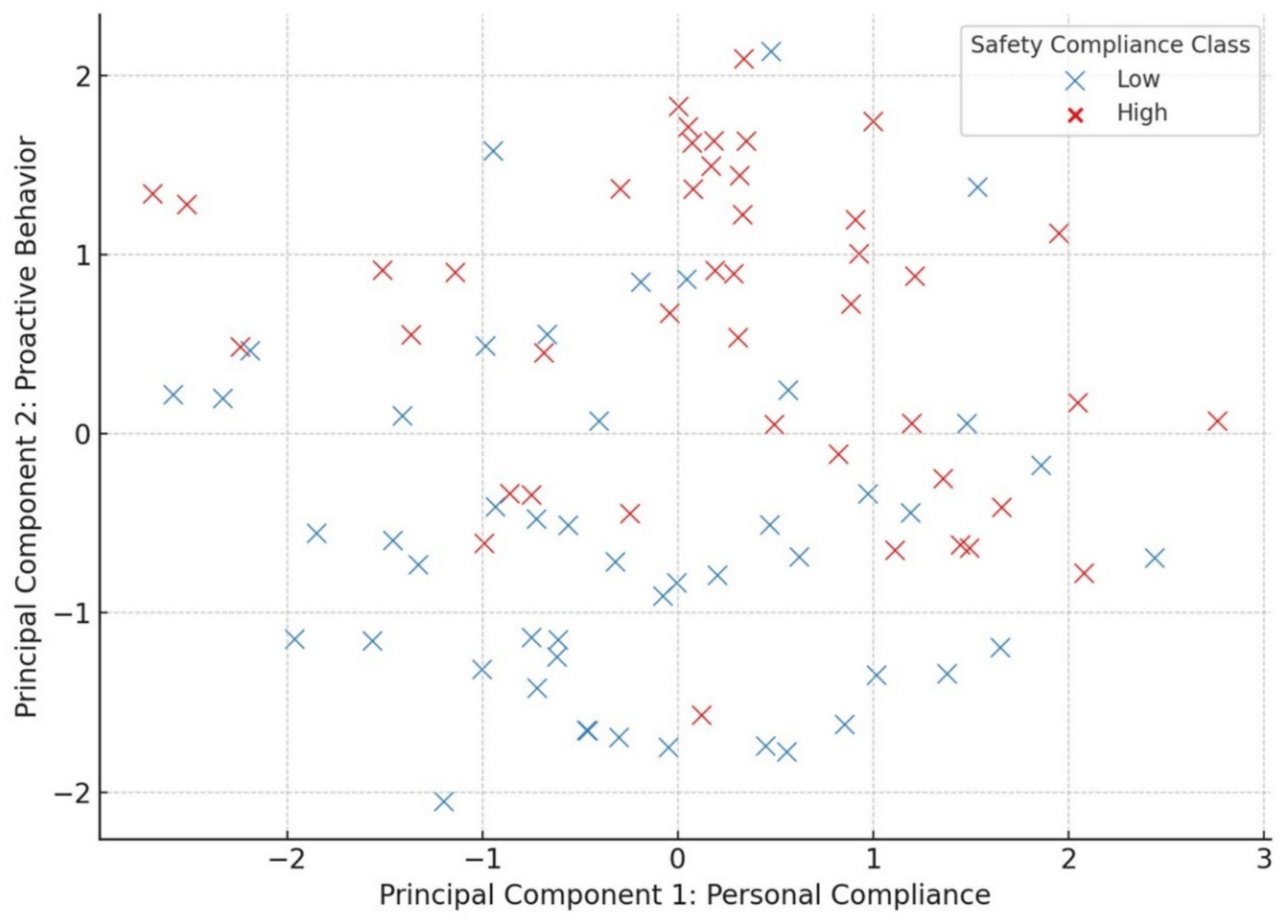
Participant distribution by PCA dimensions.

In addition, a two-dimensional visualization of participants based on the first two principal components is provided in Figure 1, where individuals are color-coded by safety behavior group (as later determined via k-NN classification).

### 3.4 Regression analysis

Multiple regression examined predictors of safety behavior. The overall model was significant, *F*(2, 89) = 39.2, *p* < 0.001, explaining 47% of the variance (*R*^2^ = 0.47, adjusted *R*^2^ = 0.46).

- Perceived institutional support was the strongest predictor (standardized *β* = 0.51, 95% CI [0.35, 0.67], *p* < 0.001, Cohen’s *f*^2^ = 0.27).
- Training exposure also predicted safety behavior (standardized β = 0.39, 95% CI [0.22, 0.55], p < 0.001, Cohen’s *f*^2^ = 0.18).

Both predictors demonstrated medium-to-large effect sizes, underscoring their substantive role in shaping safety behavior. The coefficients and model summary are presented in Table 3.

**Table 3:** Regression coefficients and model summary.

Predictor	*B*	SE	%95 Cl (lower, upper)	*β*	*t*	*p*
Perceived institutional support	2.51	0.34	[1.84, 3.18]	0.62	7.39	<0.001
Training exposure	1.84	0.29	[1.27, 2.41]	0.48	6.34	<0.001

**Figure 2:**
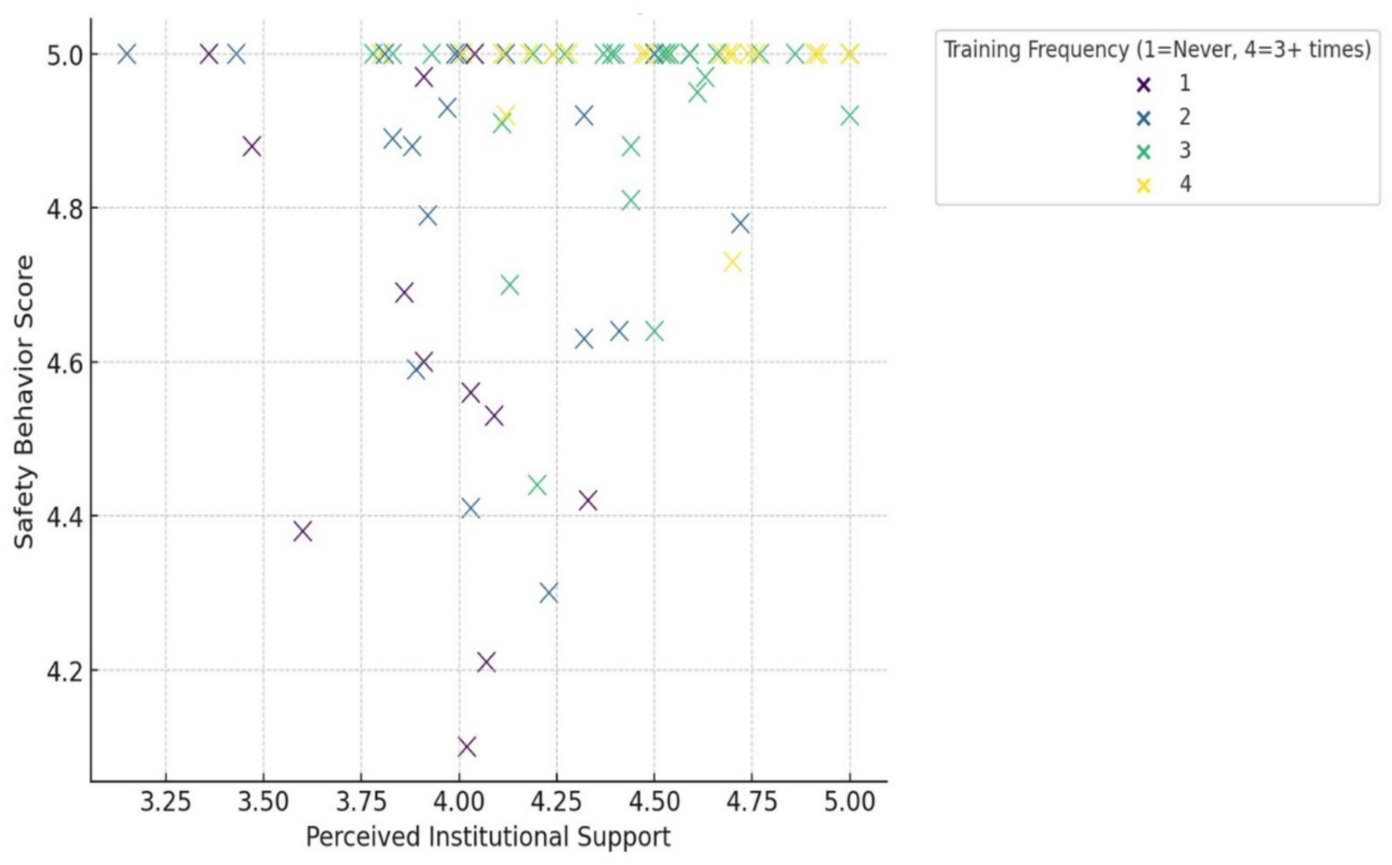
Relationship between institutional support and safety behavior stratified by training frequency.

In addition to the regression coefficients presented in Table 3 and Figure 2 visualizes the relationship between perceived institutional support, training frequency, and safety behavior scores. The scatterplot illustrates a clear positive trend, particularly among participants with frequent training exposure and high organizational support.

This visualization illustrates the relationship between perceived institutional support and safety behavior scores, color-coded by training frequency. The positive linear trend suggests that higher institutional support is associated with higher safety behavior scores across all training groups. Notably, participants with frequent training (coded as 4) cluster in the upper-right quadrant, indicating consistently high compliance when both predictors are favorable.

### 3.5 Regression diagnostics

Model assumptions were evaluated through residual and multicollinearity checks. Variance Inflation Factor values were below 1.5, indicating low multicollinearity. Standardized residuals were normally distributed and homoscedastic, as confirmed by Q–Q plots and residual scatterplots. The Durbin–Watson statistic was 1.94, suggesting no autocorrelation in residuals.

### 3.6 K-NN classification

k-NN analysis (*k* = 5, Euclidean distance, five-fold cross-validation) classified participants into two clusters: high vs. low compliance. Classification accuracy was 88%, with supporting metrics of precision = 0.86, recall = 0.84, and F1 = 0.85. This indicates that the model reliably distinguished between different behavioral profiles despite the modest sample size.

The distribution of participants across the two classes is summarized in Table 4.

**Table 4:** Participant distribution by safety behavior class (k-NN).

Class	Number of participants
Low safety behavior	45
High safety behavior	47

**Figure 3:**
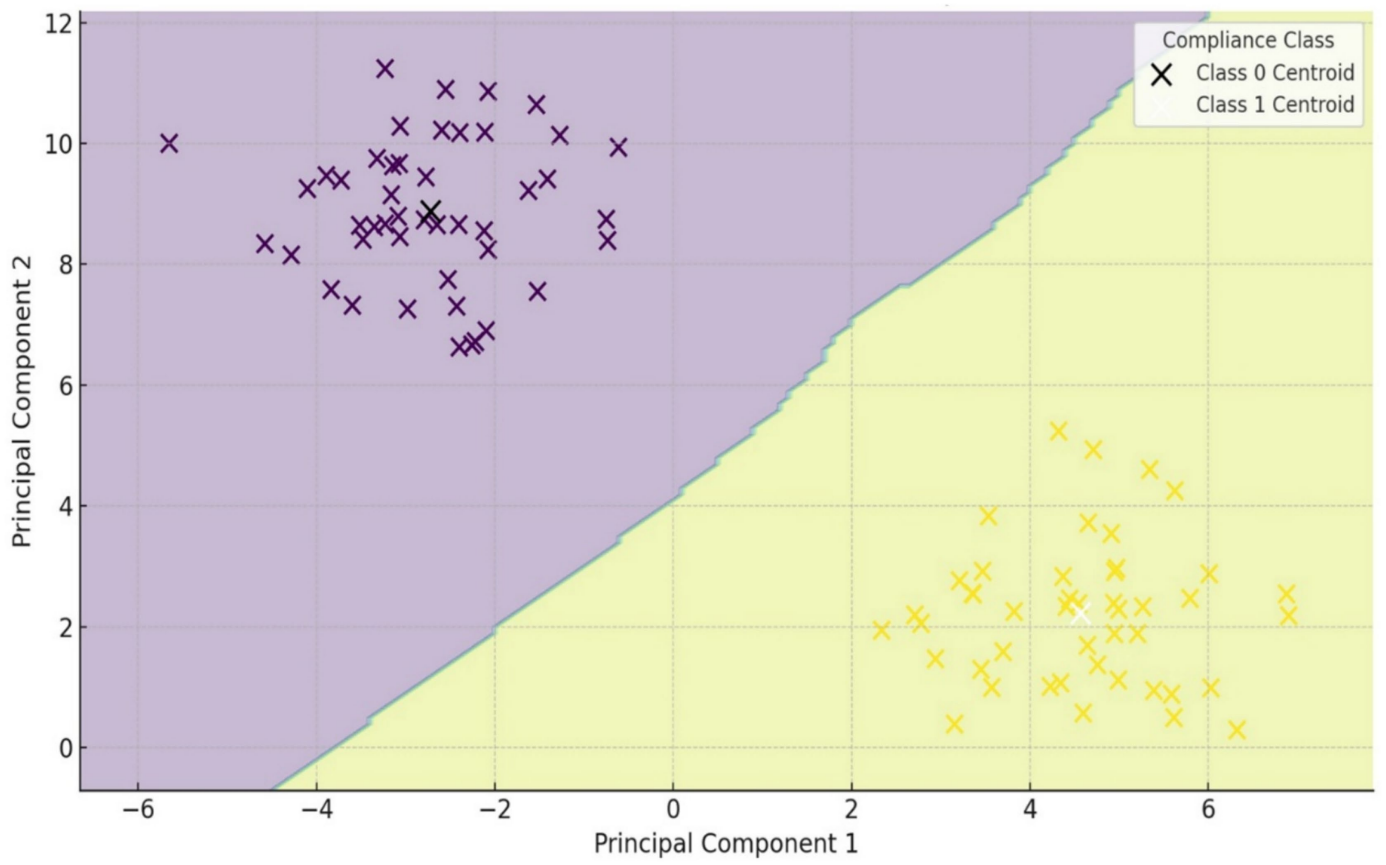
K-NN classification of safety behavior profiles.

Figure 3 presents an enhanced visualization of the classification output. The plot shows the distribution of individuals across the first two PCA components, marked by their k-NN-derived class membership. Class centroids are displayed as ‘×’ markers, while ellipses illustrate the behavioral variance and boundaries of each compliance group. This representation highlights the behavioral divergence between high- and low-compliance clusters in laboratory safety behavior.

## 4 Discussion

This study demonstrates that institutional support and safety training are robust predictors of laboratory safety behavior in genetic diagnostic settings. The use of PCA and k-NN provided a novel means of classifying safety profiles, enabling tailored behavioral insights. These findings extend prior literature while offering context-specific contributions to understanding occupational safety in Türkiye (10).

Unlike many international studies, the present research highlights the influence of local organizational dynamics. In Istanbul-based laboratories, safety culture is shaped not only by technical standards but also by hierarchical structures, resource allocation, and managerial priorities. For example, in contexts where productivity is prioritized over compliance, staff may underreport unsafe practices despite training (11). Cultural norms—such as high respect for authority and reluctance to challenge supervisors—may also reduce proactive reporting of risks, highlighting a need for policies that encourage open dialogue without fear of retribution (12).

The multidimensional structure of safety behavior identified through PCA (personal compliance, proactive behavior, institutional engagement) confirms that safety is not a unitary construct but an outcome of interactions between individual practices and organizational systems. Regression findings further reinforce the pivotal role of institutional support and structured training, aligning with international evidence while underscoring their critical importance in Türkiye’s laboratory context, where safety infrastructure may be unevenly distributed (13, 14).

The application of k-NN classification revealed heterogeneity in safety behavior, dividing employees into low- and high-compliance groups. While this provides a practical framework for targeted interventions, it also raises important ethical considerations. Behavioral profiling, if misused, risks stigmatizing individuals or creating inequities in professional development opportunities (15). Ethical safeguards must therefore accompany the use of profiling techniques, including anonymization, transparent communication of purpose, and ensuring that classification outcomes are used to support rather than penalize employees. Embedding these methods within a framework of fairness and institutional accountability is essential for maintaining trust.

From a methodological perspective, the integration of behavioral theory with statistical and computational methods strengthens the case for multidimensional and data-driven safety management. However, limitations must be acknowledged. The reliance on self-reported data introduces potential social desirability bias, and the sample was restricted to Istanbul, which may limit generalizability to other cultural or institutional contexts (16). The modest sample size for PCA also restricts the stability of extracted components. Moreover, while k-NN achieved high accuracy, its sensitivity to noise requires cautious interpretation and complementary approaches in future research.

### 4.1 Practical implications

The classification of laboratory personnel into safety profiles provides institutions with actionable tools for tailoring interventions. Low-compliance employees may benefit from targeted coaching, structured mentorship, or environmental redesign, whereas high-compliance individuals can serve as peer role models. Importantly, institutional leaders should integrate these interventions within existing cultural and managerial frameworks—balancing accountability with support. Embedding behavioral analytics into digital safety dashboards could allow for continuous monitoring, provided that safeguards are in place to protect confidentiality and ethical standards (17).

### 4.2 Study limitations and future directions

Despite its contributions, this study has several limitations. First, the cross-sectional design restricts causal inference, and the Istanbul-based sample limits generalizability beyond its cultural context. Second, while PCA revealed clear behavioral dimensions, alternative approaches such as exploratory factor analysis or confirmatory factor analysis may provide additional robustness. Third, reliance on self-reports increases the risk of social desirability bias; future studies should incorporate supervisor ratings, direct observation, or sensor-based monitoring (18). Fourth, while k-NN classification demonstrated utility, future research should compare results across multiple algorithms and include ethical safeguards to prevent stigmatization (19). Finally, expanding the model to include psychosocial constructs such as moral disengagement, risk perception, or cultural safety norms could deepen explanatory power and practical relevance.

## 5 Conclusion

This study provides compelling evidence that laboratory safety behavior is a multidimensional construct shaped by personal routines, proactive practices, and institutional engagement. Using a behaviorally grounded analytic approach, we demonstrated that perceived organizational support and training exposure significantly enhance employees’ commitment to safety. By applying principal component analysis and k-nearest neighbors classification, we identified distinct behavioral profiles that can inform targeted safety interventions. These findings highlight the value of integrating applied behavior analysis with data-driven classification techniques in occupational settings, suggesting that organizations should adopt differentiated strategies responsive to behavioral variability rather than relying on uniform safety protocols. Behaviorally anchored classification tools, as introduced in this study, represent a promising avenue for fostering personalized and sustainable improvements in safety culture. As laboratory environments—especially genetic diagnostic settings—continue to grow in complexity and risk, prioritizing behavior-based safety approaches will be essential to achieving not only regulatory compliance but also meaningful, lasting reductions in workplace hazards.

## Data Availability

The raw data supporting the conclusions of this article will be made available by the authors, without undue reservation.
